# Tibial tubercle avulsion fractures in children and adolescents

**DOI:** 10.1002/pdi3.2521

**Published:** 2025-03-12

**Authors:** Hailun Yao, Yuanlin He, Xiang Li, Mingyan Shi, Peikang Wang, Man Zhang, Xinkai Zhang, Xing Liu

**Affiliations:** ^1^ Department of Orthopedics Ministry of Education Key Laboratory of Child Development and Disorders National Clinical Research Center for Child Health and Disorders China International Science and Technology Cooperation Base of Child Development and Critical Disorders, Chongqing Key Laboratory of Pediatrics Children's Hospital of Chongqing Medical University Chongqing China; ^2^ West China School of Medicine Sichuan University Chengdu China

**Keywords:** growth plate, pediatric, skeletal maturity, tibial tubercle avulsion fractures (TTAFs)

## Abstract

Tibial tubercle avulsion fractures (TTAFs) are uncommon injuries in the pediatric population, predominantly affecting children and adolescents who are approaching skeletal maturity and frequently engage in high‐energy activities. Despite of their rarity, TTAFs can significantly impact the lives of young individuals involved in sports and other strenuous activities. The mechanism of TTAFs occurrence involves forceful quadriceps contraction against resistance or rapid knee flexion with contracted quadriceps. TTAFs may coincide with other related injuries due to their mechanism of occurrence and commonly present with an abrupt onset of pain, focal soft‐tissue swelling and tenderness on palpation. Predisposing factors such as a history of Osgood‐Schlatter disease and an extreme body mass index (BMI) could contribute to TTAFs susceptibility. Diagnosis of TTAFs typically relies on X‐rays, complemented by computed tomography (CT) and magnetic resonance imaging (MRI) for screening associated injuries and preoperative assessment. While a well‐established classification system exists, with the Ogden classification being the most commonly employed, intriguingly, a direct correlation between fracture type and treatment method, as well as the choice of surgical fixation modality, remains elusive. The management of TTAFs encompasses both conservative and surgical approaches, with open reduction internal fixation (ORIF) being the predominant surgical method and the prognosis for this condition is generally favorable. By synthesizing existing knowledge and presenting potential areas of uncertainty, this review aims to offer valuable insights to orthopedic practitioners when they are confronted with this infrequent injury.

## 1 INTRODUCTION

**FIGURE 1:**
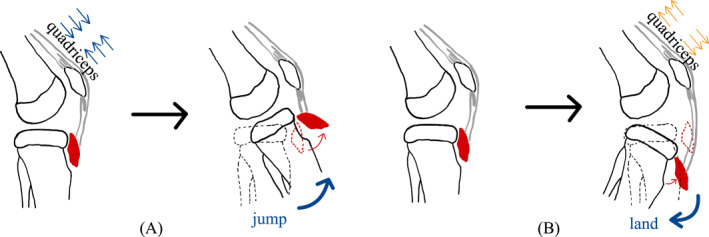
(A) Quadriceps contraction during jumping causing proximal tibial avulsion fracture. (B) Forced quadriceps stretch during landing causing proximal tibial avulsion fracture.

Tibial tubercle avulsion fractures (TTAFs) are rare injuries, accounting for 0.4%–2.7% of pediatric fractures and <1% of all physeal injuries.^1^, ^2^ However, the prevalence of proximal tibial fractures is rising as a result of increased participation of youth in high‐energy activities.^3^, ^4^ TTAFs are frequently observed in adolescents with an average age of 14.6 years and males are more likely to sustain such injuries than females.^5^, ^6^, ^7^ Male individuals are considered to be at a heightened risk compared to females, a trend attributed to several factors. Notably, males tend to possess greater quadriceps strength than females, leading to increased stress on the cartilage.^8^ Additionally, the later closure of the proximal tibial physis^9^ and a higher rate of participation in athletic activities further contribute to this elevated risk in males. In contrast, females exhibit a higher propensity for low‐grade Ogden type fractures (*p* < 0.004)^10^ when compared to males. This specific fracture type commonly arises during sports involving repetitive jumping (e.g., basketball, football), situations where the quadriceps are forcefully contracted against resistance, such as during jumping (Figure 1A), or when rapid knee flexion occurs with contracted quadriceps, such as during landing (Figure 1B).^2^, ^6^, ^11^, ^12^

According to the systematic review in 2016, associated injuries occur in approximately 8% of TTAF cases.^6^ The most common associated injuries are patellar tendon avulsions (2%), meniscal tears (2%) and preoperative compartment syndromes (4%). A recent large retrospective case series from 2021 has reported a higher incidence, with rates of patellar tendon ruptures (full or partial) and compartment syndrome standing at 9% and 2%, respectively.^10^ Less frequent injuries encompass periosteal damage, avulsion of the distal pole of the patella, coronary ligament injury, vascular compromise and joint laxity.^9^, ^13^, ^14^, ^15^ It is vital to recognize the compartment syndrome due to its potential catastrophic outcomes. The rate of compartment syndrome has been reported to be as high as 20%,^13^ while more recent research points to a rate of approximately 2%–4%.^6^, ^10^ The rates of compartment syndrome vary widely between newer and earlier studies, which is possibly owing to previous selection, publication bias and undiagnosed cases. Additionally, in a more recent study, Haber et al. found that compartment syndrome occurs in type IV fractures around five times more frequently than that in other kinds (*p* < 0.05), and theorized that type IV and V fractures are both at a higher risk because of the extension through the physis into the posterior tibia.^10^ This review will comprehensively address various facets of TTAFs, encompassing predisposing factors, pathophysiological mechanisms, clinical manifestations, imaging modalities, classifications, treatment options, common complications, and the disease prognosis in following parts. Through the synthesis of existing knowledge and identification of potential areas of uncertainty, this review aims to provide valuable insights to orthopedic practitioners when they encounter this relatively uncommon injury.

## 2 PREDISPOSING FACTORS

The potential link between Osgood Schlatter disease (OSD) and TTAFs has been suggested.^16^ In the most recent systematic review conducted in 2016, approximately 25% of patients exhibited OSD symptoms during the review period.^6^ However, it is important to note that a direct cause‐and‐effect relationship between OSD and TTAFs has not yet been definitively established, and the current understanding suggests an association rather than a causative connection.^2^, ^6^, ^16^, ^17^, ^18^, ^19^ A recent retrospective comparative study has shown that patients with both tibial tubercle fractures and OSD tend to have higher posterior tibial slope angles (PTSAs) compared to the control group.^20^ This observation lends support to the notion that an elevated tibial slope places the proximal tibial physis under atypical stress, potentially accelerating the development of pathological conditions in the proximal tibia, including OSD and tibial tubercle fractures. Controversy also exists regarding the fracture types of TTAFs in patients with OSD. Pretell‐Mazzini et al. discovered that 62% (18/29) of the reviewed OSD patients had type III TTAFs. Importantly, their systematic review indicated that the differences in OSD occurrence among various fracture types were not statistically significant (*p* = 0.641)^6^, while in a large retrospective case series, Haber et al. reported that occurrence of OSD tends to be associated with type I fractures (46%, *p* < 0.001).^10^

An abnormal body mass index (BMI) has also been proposed as a potential risk factor.^10^, ^21^, ^22^, ^23^ Shin YW et al. have indicated that an extreme BMI might pose a risk for tibial tubercle avulsion fractures in adolescents, particularly during low‐energy activities such as running, even in the absence of clearly defined trauma. This inference is based on their study involving the evaluation of 30 patients with TTAFs.^22^ Haber et al. have hypothesized that BMI could potentially impact both the treatment and prognosis of the fracture. Their findings reveal that mean BMI *Z*‐scores are significantly higher for fractures treated surgically compared to those managed non‐surgically (*p* = 0.05).^10^ Additionally, within the lower BMI group (BMI < 20), a noteworthy 75% of patients reported pain with squatting, as opposed to 18%–32% of patients in higher BMI categories (*p* = 0.002). Presenting a fresh perspective, Shin et al. has underscored that the primary risk factor may be an inappropriate knee extensor mechanism, rather than solely an extreme BMI.^22^ For example, they point to factors like a weakened physis due to abnormal loading in obese children, diminished bone strength linked to bone mineral density in underweight children,^24^, ^25^ or heightened extensor power in relation to body weight compared to physeal strength.^22^ However, Sheppard et al. dispute the connection between BMI and TTAFs.^20^ In their study, while BMI exhibited significant differences between groups (*p* = 0.003), a more precise parameter in children, the BMI percentile, did not exhibit significant differences. Despite the exact causal relationship remaining unidentified, Riccio et al. observed no discernible disparity in functional outcomes of the fracture based on BMI^26^ ^.^

Further research is required to determine whether there are causative relationships between OSD/BMI and TTAFs.

## 3 PATHOPHYSIOLOGY

The pathophysiology of TTAFs is intricately linked to the pattern of ossification within the knee joint. The proximal tibia features two ossification centers—a primary site situated at the proximal tibial physis and a secondary site located at the tibial tubercle or apophysis. The ossification of the tibial tubercle progresses through four stages: the cartilaginous stage, the apophyseal stage, the epiphyseal stage, and the osseous stage.^27^ The cartilaginous stage appears before the secondary ossification center manifests in the tubercle. The apophyseal stage begins with the development of the secondary ossification center, which typically takes place at the ages of 8–12 years in girls and the ages of 9–14 in boys.^28^ Histologically, fibrocartilage, as opposed to columnar cartilage cells, makes up almost all of the physis beneath the tuberosity, which resists the tensile forces applied by the patellar tendon throughout skeletal growth.^27^ During the apophyseal stage, the tibial tubercle and the secondary ossification center of the proximal tibia combine to form an anterior tongue of bone in continuity with the proximal tibial epiphysis. The proximal tibial physis closes in the posteromedial to anterolateral direction.^28^ The fibrocartilage is gradually replaced from proximally to distally by columnar cartilage, which is susceptible to fail under tensile stress, as the secondary ossification center of the tuberosity matures. The alterations generate a zone that is mechanically two to five times weaker than the surrounding fibrous connective tissue and predisposed to avulsion in the tibial tubercle.^1^, ^29^ The epiphyseal stage occurs at the ages of 10–15 years in girls and the ages of 11–17 years in boys. By the time girls reach the age of 15 and boys reach the age of 17, the proximal aspect of the tibia and the ossified tuberosity have normally fused together, resulting in a unified structure (Table 1).

**TABLE 1:** Stages of tibial tubercle ossification.

The ossification stage	Age (girls)/year	Age (boys)/year
Cartilaginous	< 8	< 9
Apophyseal	8–12	9–14
Epiphyseal	10–15	11–17
Osseous	15+	17+

**FIGURE 2:**
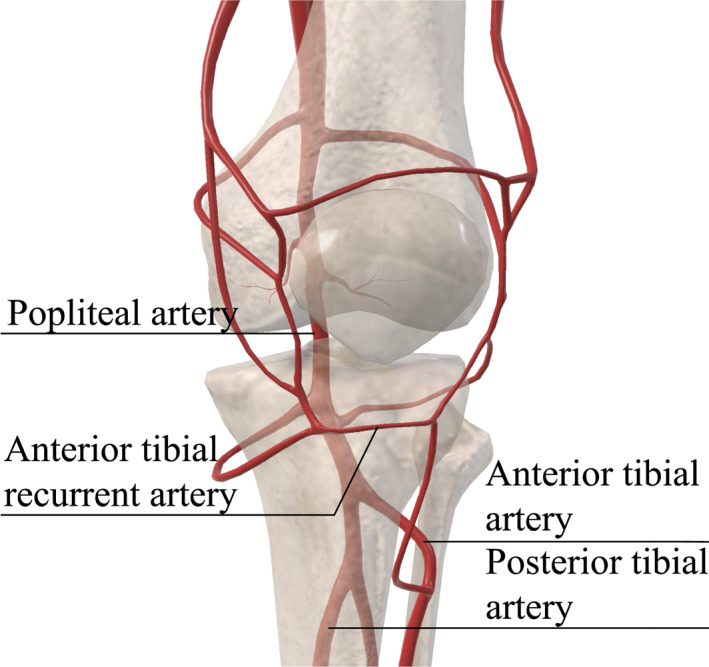
A schematic illustrates the relationship between tibial tubercle fracture and the vascular system. The proximal tibia is in close proximity to the popliteal artery which adds the possibility of the entrapment syndrome. The anterior tibial recurrent artery is associated with the incidence of compartment syndrome.

Furthermore, tibial tubercle fractures entail a notable susceptibility to compartment syndrome, largely attributed to the unique anatomy of the proximal tibia and the tibial tubercle. In cases of displaced tubercle fractures, there exists a potential for harm to the anterior tibial recurrent artery, which arises superiorly above the tibial tubercle^30^ (Figure 2).

## 4 CLINICAL MANIFESTATION AND DIAGNOSIS

TTAFs often occur during jumping and landing, and commonly present with an abrupt onset of pain, focal soft‐tissue swelling and tenderness on palpation at the anterior portion of the tibial tubercle, as well as the inability to move the knee, load and ambulate. Although TTAFs are rare injuries, plain radiographs of the knee are generally sufficient for making a diagnosis. However, they tend to underestimate the extent of intra‐articular involvement. As a result, cross‐sectional imaging, such as computed tomography (CT) or magnetic resonance imaging (MRI), is highly recommended to provide precise guidance for definitive management. More information about imaging will be presented in subsequent section 5: **Imaging**. Given the distinct clinical presentation and accurate imaging, the diagnosis becomes evident, thereby often eliminating the need for differential diagnosis. Moreover, conducting a comprehensive neuromuscular examination is crucial due to the elevated risk of compartment syndrome development and, potentially, vascular injury associated with this fracture type. Physical examinations frequently reveal edema, effusion (hemarthrosis in Ogden type III fractures), and ecchymosis. Since the anterior tibial tubercle and tibial plateau are easy to palpate owing to the minimal soft tissue coverage, it is easy but crucial to identify the specific regions of pain and tenderness.^3^, ^6^ However, the retinacular fibers, supported by robust periosteal coverage, can facilitate active extension in Ogden type II and III fractures. It is noteworthy that these fracture types often exhibit an inadequate extensor mechanism. It is also essential to evaluate knee stability through specialized tests, including varus/valgus stress, anterior/posterior drawer, and pivot‐shift maneuvers. Evaluation of the vascular system is indispensable due to the anatomical proximity to the popliteal artery and the possibility of developing the entrapment syndrome and compartment syndrome which can cause catastrophic effects when there is a delay in treatment^31^ (Details are described in the section 3: **Pathophysiology**, Figure 2).

## 5 IMAGING

Initial imaging should include anteroposterior and lateral knee radiographs. The lateral radiograph, in particular, has been conventionally employed to assess the quality of reduction across diverse fracture patterns, injury classifications, and treatment recommendations. This lateral view excels in depicting the position of the patella and facilitates comparison with the contralateral knee. For a comprehensive assessment of fracture extent and potential fragmentation, particularly concerning intra‐articular involvement and posterior extension, preoperative CT scans offer significant assistance. Nonetheless, the utilization of CT is constrained by its radiation exposure, especially concerning pediatric patients, and its limited capability in detecting soft tissue injuries. In contrast, MRI stands as the optimal modality for evaluating soft tissue injuries like ligamentous or meniscal tears. Yet, the drawbacks of MRI include the absence of comprehensive bony details regarding the fracture pattern, along with the associated cost and lengthy scan duration.

## 6 CLASSIFICATION

**FIGURE 3:**
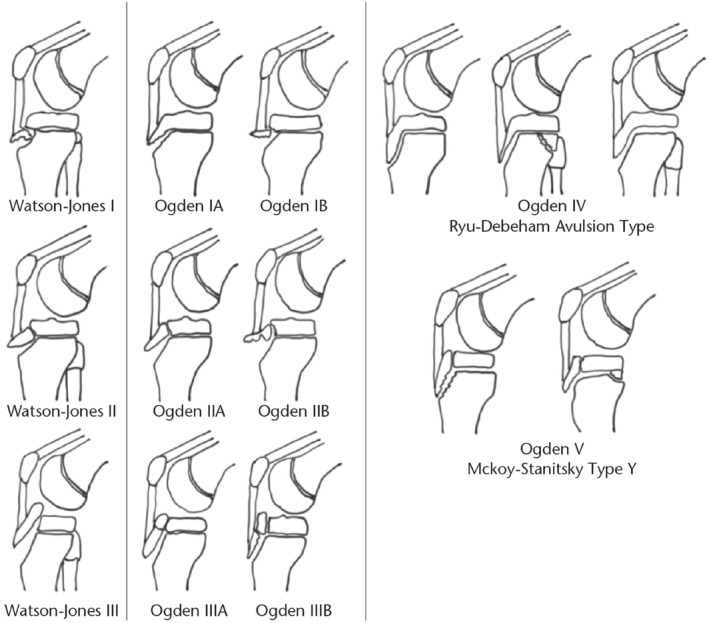
The Ogden, Watson‐Jones, Ryu, and McKoy classifications and modifications of tibial tubercle avulsion fractures.

The most widely used technique for classifying TTAFs in clinical practice is the Ogden, Watson‐Jones, Ryu, and McKoy classification and modification system of tibial tubercle avulsion fractures (Figure 3). Watson‐Jones first established the original classification in 1955, which comprised fractures type I through III. Type I fractures characterize avulsions of the tibial tubercle distal to the proximal tibial physis. Type II fractures include extension across the physis but do not enter the knee joint, and type III fractures are avulsions which extend proximally to the physis and into the knee.^32^ Then in 1976, Ogden and colleagues added A and B modifications, completing the classification system that is still in use today. Then, a type IV classification fracture was described as extending posteriorly along the entire proximal tibial physis with the potential to separate the entire epiphysis.^33^ In 1990, ligament avulsion injuries from the patella were proposed as type C modifications.^34^ Following that, a type V fracture was classified, which is a type IIIB with a concurrent Salter–Harris type IV fracture that results in a distinctive Y configuration.^28^

**FIGURE 4:**
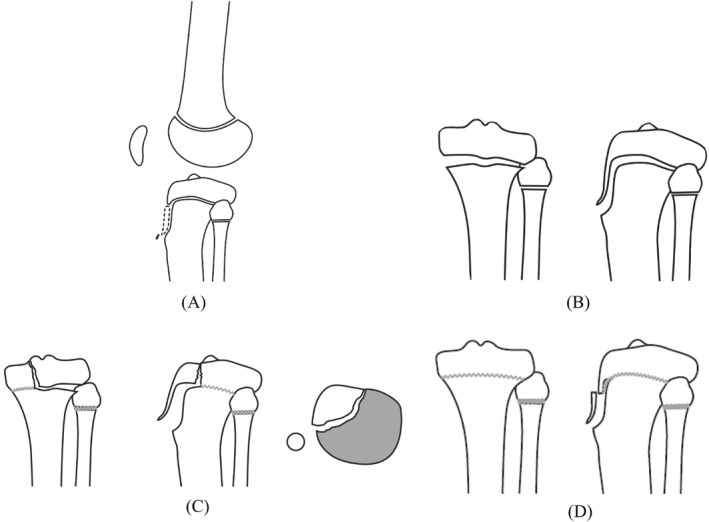
The Pandya classification system.

Although the classic classification and modifications encompass all spectra of TTAFs, they grossly understate the extent of intraarticular involvement. In 2012, Pandya et al. proposed a new classification with four types: type A is isolated fractures of the ossified tip, type B is injuries occurring when the epiphysis and tubercle fracture as a unit off the metaphysis without intra‐articular involvement, type C fractures extend to the intra‐articular surface of proximal tibia, and type D fractures only involve the distal aspect of the tubercle (Figure 4).^35^ CT may help to delineate intra‐articular involvement or extension of the fracture into the metaphysis. MRI may be useful to identify meniscal tears, osteochondral defects, associated patellar or quadriceps tendon rupture. However, the value of routine use of advanced imaging or arthroscopy remains questionable. Brown et al. found that the CT did not contribute to correct fracture classification, nor did it reclassify any fracture to a modified Ogden IV or V and supplemented that the intraarticular injury can be identified during the direct visual inspection of the knee joint.^36^ In a retrospective case series of TTAFs, 25 fractures underwent CT for further evaluation, 25 fractures were performed to receive further evaluation. CT changed the Ogden fracture classification for 6 (24%) fractures and five of them resulted in a higher Ogden fracture class.^10^ More reports are needed to develop evidence‐based recommendations on the need for CT/MRI for further evaluation.

More recently, Yousef has highlighted a critical concern regarding the commonly employed classification system, which primarily focuses on the tibial tubercle fracture pattern, while overlooks the extent of associated tendinous injuries. In his study, Yousef shed light on the limitations of the Ogden classification, emphasizing its lack of prognostic value due to the absence of significant functional outcome disparities among various fracture types. Consequently, he introduced a novel classification system that takes into account both the fracture characteristics and associated tendinous injuries.^18^

## 7 TREATMENT

**FIGURE 5:**
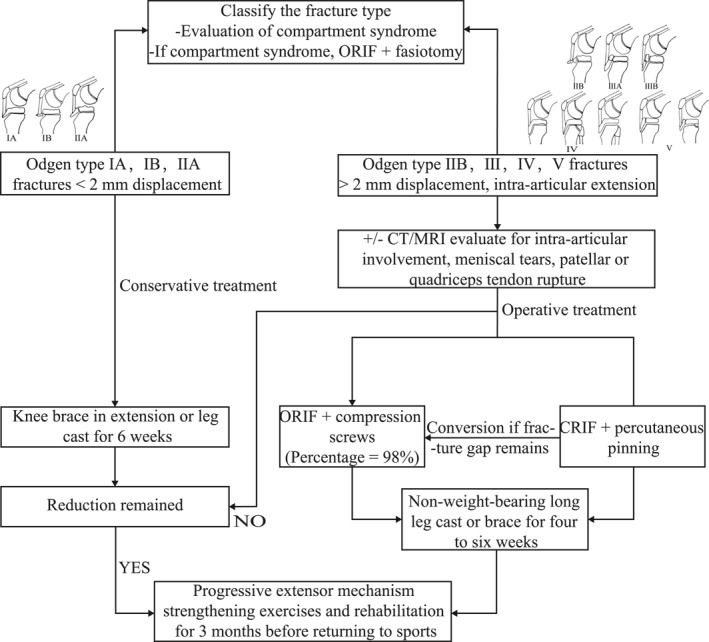
Suggested treatment algorithm for TTAFs. CRIF, closed reduction and internal fixation; ORIF, open reduction internal fixation.

The objectives of treatment are to preserve the tibial articular surface congruency, achieve an anatomic reduction of the fracture fragment and restore the extensor mechanism. Despite a well‐established classification system, there are currently no evidence‐based clinical practice guidelines for surgical or nonsurgical treatment of TTAFs as well as the modality for surgical fixation which is largely up to the orthopedic surgeons. A study conducted in 2022 has partly shown the significant variability among surgeons when evaluating and treating pediatric tibial tubercle fractures.^37^ The flexibility in treatment options likely emerges from a combination of factors, including the relative rarity of TTAFs and the favorable prognosis associated with these fractures, irrespective of whether they are managed conservatively or through surgical intervention.^11^ Some authors recommend that the type IA, IB, and IIA fractures are managed by conservative treatment, and the type III, IV and V fractures are more suitable for operative treatment.^9^, ^16^, ^38^, ^39^ But in the most recent systematic review,^6^ Pretell‐Mazzini et al. pointed out that no correlation was found between fracture types and treatment methods and postulated that the severity of displacement and associated injuries are more important factors of treatment than the location of the fracture line. The research conducted by Checa Betegón et al., which adds to the evidence supporting the postulation, describes a series of five type IV fractures that were treated non‐surgically and all five returned to sports in less than 25 weeks.^19^ A suggested treatment algorithm for TTAFs is presented in Figure 5.

### 7.1 Nonoperative treatment

Some authors advocate conservative management for fractures categorized as Type IA, IB, or IIA.^9^, ^16^, ^38^, ^39^ Conversely, other authors propose that nonoperative treatment is warranted for fractures with no displacement or displacement less than 2 mm, along with achieving satisfactory alignment in the cast after closed reduction and cast immobilization.^16^, ^40^ However, the indications for nonoperative treatment are not standardized. A knee brace in extension or a long leg cast for 6 weeks is used as a conservative treatment to immobilize the knee in extension. Patients who undergo conservative treatment require vigilant monitoring.^9^ Repeat knee radiographs should be obtained at intervals of 7–10 days, 3 weeks, and 6 weeks post‐injury. These radiographs serve the purpose of evaluating whether any distal fragment displacement has occurred as swelling subsides. If an adequate reduction is not achieved, conversion to surgical management is recommended.

### 7.2 Operative treatment

There exists no standardized criterion for surgical intervention either. Certain authors advocate for surgical intervention when the fracture type is IIB, III, IV, or V. Conversely, some experts propose that operative treatment is warranted for fractures exhibiting displacement of ≥ 2 mm, intra‐articular extension causing joint incongruity, and for patients who cannot tolerate non‐weightbearing in a cast.^40^ Notably, a systematic review conducted in 2016 indicated that surgical treatment was employed in 88% of TTAF cases. Surgical options include open reduction internal fixation (ORIF) with compression screws, closed reduction and percutaneous pinning and additional interventions include fasciotomies for compartment syndrome and arthroscopic assistance. While various surgical choices are available, open reduction and internal fixation are the most frequently applied methods, accounting for 98% of cases.^6^

ORIF consists of open reduction and internal fixation. Open reduction can be achieved by either direct arthrotomy or arthroscopic view. The former method holds an advantage in addressing fractures accompanied by soft tissue injuries and intra‐articular extension. However, a drawback of arthrotomy is that it extends the duration of both immobilization and rehabilitation. In the case of arthroscopy, it is imperative to acknowledge the technical complexities involved and the heightened risk of compartment syndrome in tibial plateau fractures.^41^ Another potential post‐arthroscopy issue is arthrofibrosis.^6^ In order to achieve complete anatomical reduction of the fracture fragments, it is vital to accomplish complete anatomical reduction of the fracture fragments, immediate examination and debridement of the fracture site, as well as the removal of any soft tissue interposition, primarily the periosteum.^31^ For effective compression of the fracture lines during internal fixation, the use of cancellous partly threaded screws is recommended. Ideally, the utilization of 4.0 mm screws is advised, as this choice helps minimize the potential for enduring soft tissue discomfort that larger screws could induce. For patients with more than 3 years of potential growth left, surgeons may also choose Kirschner wires, but biomechanical studies have shown that cannulated screws offer superior fixation comparing with Kirschner wires.^3^ In the context of choosing between unicortical and bicortical fixation, it's important to note that unicortical fixation offers the benefit of safeguarding the posterior neurovascular structures and avoids penetrating through the posterior cortex. On the other hand, bicortical fixation offers the advantage of creating a sturdier construct, although this advantage comes with the potential drawback of increased risk to the posterior neurovascular systems.^42^ Arkader et al. observed that the outcome of Odgen type I to III fractures was not significantly different between unicortical and bicortical fixation and recommended the use of unicortical fixations for these fracture types to mitigate the potential risk of vascular injuries.^42^ Additionally, in order to avoid vascular injury to the popliteal artery caused by the screw fixation, Frederick Mun et al. recommend directing screws within the medial 60% of the tibia after characterizing the variation of the popliteal artery among pediatric patients.^43^ This discovery supports the use of bicortical fixation in certain fracture patterns, such as Odgen type IV fractures which often demand additional support and rigidity offered by bicortical fixation.

Arthroscopy is particularly indicated for TTAFs when there is intra‐articular involvement, especially with fracture displacement. This technique facilitates precise evaluation and repair of the joint surface, as well as assessment and management of associated meniscal or osteochondral injuries. However, it is crucial to mind the risk of compartment syndrome, which can be minimized by limiting the duration of arthroscopy and opting for either dry or with a low‐pressure bomb.^35^, ^39^, ^40^, ^44^ Postoperative arthrofibrosis is also a potential concern that should be monitored.^6^

Closed reduction and internal fixation (CRIF) has often been overlooked in the surgical treatment of TTAFs. However, Jardaly et al. conducted a study and recommended that CRIF could be initially attempted for tibial tubercle fractures. They discovered that closed reduction with internal fixation (CRIF) represents a less invasive treatment strategy for these fractures. CRIF offers advantages such as reduced bleeding, avoidance of tourniquet use, and shorter operative durations and has identical complication rates and prognosis when compared to ORIF.^45^ The main argument against closed reduction is the necessity to reveal associated soft tissue injuries, but given the rarity of the soft tissue injuries (details are described in the **Associated Injuries** above) and the most frequent injuries such as patellar tendon and the ability to tentatively diagnose common injuries like patellar tendon issues and compartment syndrome before surgery, CRIF could be initially attempted for tibial tubercle fractures. However, if a fracture gap persists after closed reduction, a low threshold for conversion to ORIF is indicated because this frequently denotes entrapped soft tissue.^46^ Furthermore, it is strongly advised to maintain a low threshold for prophylactic fasciotomy. This approach is warranted due to the severe consequences associated with delayed or overlooked diagnosis and treatment of compartment syndrome, which encompass nerve injury, muscle necrosis, and vascular compromise.^10^, ^15^, ^36^, ^47^ Diagnosis can be made based on patients' symptoms, intra‐compartmental pressure, or combined. Nonetheless, it's noteworthy that Brown et al. found that prophylactic fasciotomy does not appear necessary in Ogden I to III TTAFs and recommended that the prophylactic fasciotomy should not be routinely used in these types of TTAFs. Instead, their recommendation is to consider performing fasciotomy when clear signs and symptoms of compartment syndrome are present.^36^

After the operation, it is advised to protect the knee for 4–6 weeks with a non‐weight‐bearing long leg cast or brace. During this phase, the focus should shift towards gradually strengthening the extensor mechanism. A 3‐month period of rehabilitation before returning to sports activities is recommended. Soft tissue restoration for “sleeve” fractures combined with periosteal sutures and an 8‐week immobilization period can be used to treat patients with relatively immature skeletons. While this technique yields a favorable prognosis for young patients, it necessitates an extended rehabilitation period to permit adequate healing of the soft tissues.^28^

## 8 COMPLICATIONS

**FIGURE 6:**
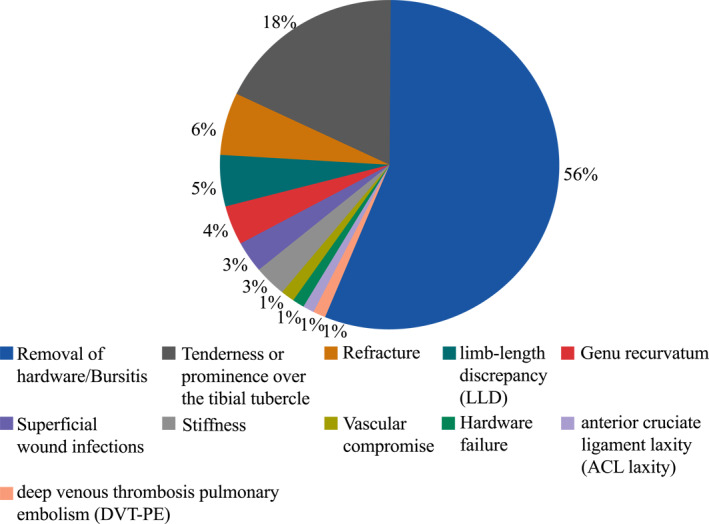
The complication rates of TTAFs.

As indicated by a systematic review conducted in 2016, the overall complication rate is estimated to be 28% (Figure 6, 95 of 336 fractures). Among these, bursitis, which required the removal of implants, was the most often reported consequence (56%), followed by tenderness or prominence over the tibial tubercle (18%). Refracture has been documented in up to 6% of cases involving type III, IV, and V fractures. Other rare complications were genu recurvatum (4%), which only affected individuals under the age of 13, superficial wound infections (3%), and limb‐length discrepancy (5%), with a mean discrepancy of 1.4 cm (range, 1–2 cm).^6^ Due to the fact that these fractures frequently take place close to skeletal maturity, growth arrest is less common than Nathan and Parikh predicted.^48^ Further elucidation of the associations between complication rates and various types of TTAFs is warranted. Franz's findings highlighted statistically significant differences in complication rates among different fracture types (*p* = 0.003).^6^ However, it is important to acknowledge that the study's sample size for type V fractures was limited, thereby necessitating additional research to conclusively establish this relationship.

## 9 OUTCOME

Pretell‐Mazzini et al. reported that 98% (250/255) of patients attained complete range of knee motion within a mean period of 22.3 weeks (range, 8–37 weeks) and 97% (248/255) of patients were able to resume to preinjury activities in a mean of 28.9 weeks (range, 12–120 weeks).^6^ Notably, no statistically significant difference was observed across various fracture types concerning the ability to return to preinjury activities (*p* = 0.163) or range of motion (*p* = 0.483), and a remarkable 99% of 336 fractures displayed union. However, due to the lack of quantitative evaluation of the functional results, Riccio et al. conducted a study to quantitatively assess the functional results of surgically managed tibial tubercle fractures in adolescents.^26^ They found that patients' thigh circumference was slightly smaller in the injured extremity (median difference, 1.7 cm at 15 cm above the patella and 4.0 cm at 50% of the length of the thigh) and 26% of patients (5/19) had a significant quadriceps extension strength deficit on the involved leg on average 31% less than the contralateral side.^26^ The findings may point to a way to modify postoperative rehabilitation in this subset of patients, aiming to improve patient‐reported outcomes.

## 10 CONCLUSION

Tibial tubercle avulsion fractures are rare injuries, accounting for 0.4%–2.7% of pediatric fractures; however, their incidence is on the rise as children increasingly engage in high‐energy sports. Further research is required to determine whether there are causative relationships between certain factors, such as OSD and BMI, and TTAFs. The pathophysiology of tibial tubercle avulsion elucidates the emergence of weak zones and the associated risk of vascular injuries. TTAFs can be easily diagnosed through clinical presentation and plain radiographs. Several classification systems have been employed to categorize these fractures; nevertheless, the Ogden classification remains the most extensively utilized and accepted. Despite the existence of well‐established classification systems, evidence‐based clinical practice guidelines are currently lacking. Treatment approaches range from nonoperative to operative, with surgical intervention, particularly open reduction internal fixation (ORIF), being the prevailing choice in most cases. Apart from the fracture itself, it is imperative to promptly rule out the occurrence of compartment syndrome, a potential early complication, and to evaluate the presence of concomitant soft tissue injuries. With timely and appropriate management, the prognosis for TTAFs is favorable, with a high rate of union observed and a return to preinjury activities.

## AUTHOR CONTRIBUTIONS

**Hailun Yao**: Methodology, data curation and writing the original draft. **Xing Liu**: Supervision, funding acquisition, review and editing. **Yuanlin He**: Visualization. **Xiang Li**: Data curation. **Mingyan Shi**: Data curation. **Peikang Wang**: Data curation. **Man Zhang**: Formal analysis. **Xinkai Zhang**: Formal analysis.

## ETHICS STATEMENT

Not applicable.

## CONFLICT OF INTEREST STATEMENT

No conflict of interest to declare.

## CONSENT FOR PUBLICATION

Not applicable.

## Data Availability

The data that support the findings of this study are openly available in Pubmed at https://pubmed.ncbi.nlm.nih.gov/?db=pubmed.
